# Effects of Exercise on Sleep Quality and Insomnia in Adults: A Systematic Review and Meta-Analysis of Randomized Controlled Trials

**DOI:** 10.3389/fpsyt.2021.664499

**Published:** 2021-06-07

**Authors:** Yi Xie, Shuai Liu, Xue-Jiao Chen, Hai-Han Yu, Yuan Yang, Wei Wang

**Affiliations:** ^1^Department of Neurology, Tongji Hospital, Tongji Medical College, Huazhong University of Science and Technology, Wuhan, China; ^2^Reproductive Medicine Center, Tongji Hospital, Tongji Medicine College, Huazhong University of Science and Technology, Wuhan, China

**Keywords:** exercise interventions, sleep quality, insomnia, Pittsburgh Sleep Quality Index, Insomnia Severity Index, actigraph, meta-analysis, RCT

## Abstract

**Study Objectives:** We conducted a meta-analysis to assess the effects of different regular exercise (lasting at least 2 months on a regular basis) on self-reported and physiological sleep quality in adults. Varied exercise interventions contained traditional physical exercise (e.g., walking, cycling) and mind–body exercise characterized by gentle exercise with coordination of the body (e.g., yoga).

**Methods:** Procedures followed the Preferred Reporting Items for Systematic Reviews and Meta-Analyses (PRISMA) guidelines. Systematical searches were conducted in three electronic databases (PubMed, Embase, and Web of Science) for relevant research that involved adult participants without pathological diseases receiving exercise intervention. The search strategy was based on the population, intervention, comparison, and outcome study design (PICOS) framework. The self-reported outcomes included varied rating scales of Pittsburgh Sleep Quality Index (PSQI), Insomnia Severity Index (ISI), and Epworth Sleepiness Scale (ESS). Subgroup meta-analyses of PSQI scores were conducted based on type of exercise, duration of intervention, and participants' age and gender. The physiological outcomes were measured by Actigraph. All meta-analyses were performed in a fixed or random statistic model using Revman software.

**Results:** Twenty-two randomized controlled trials were included in the analysis. The overall analysis on subjective outcomes suggests that exercise interventions significantly improved sleep quality in adults compared with control interventions with lower PSQI (MD −2.19; 95% CI −2.96 to −1.41), ISI (MD −1.52; 95% CI −2.63 to −0.41), and ESS (MD −2.55; 95% CI −3.32 to −1.78) scores. Subgroup analyses of PSQI scores showed both physical and mind–body exercise interventions resulted in improvements of subjective sleep to the same extent. Interestingly, short-term interventions (≤3 months) had a significantly greater reduction in sleep disturbance vs. long-term interventions (>3 months). Regarding physiological sleep, few significant effects were found in various sleep parameters except the increased sleep efficiency in the exercise group vs. control group.

**Conclusions:** Results of this systematic review suggest that regular physical as well as mind–body exercise primarily improved subjective sleep quality rather than physiological sleep quality in adults. Specifically, self-reported sleep quality, insomnia severity, and daytime sleepiness could be improved or ameliorated with treatment of exercise, respectively, evaluated by PSQI, ISI, and ESS sleep rating scales.

## Introduction

It has been widely acknowledged that sleep is an important component for people's health and wellness across the whole life (1). In recent years, insomnia has become increasingly prevalent in adults, which could inevitably bring negative effects on their daily lives and work (2, 3). Chronic insomnia contains difficulties in falling asleep, maintaining sleep, and early morning wakening (4). It is reported that ~25% of the population of the United States described insomnia complaints, and nearly 10% fulfilled the diagnostic criteria for chronic insomnia (5–7). Insomnia is not an inherent part of aging but rather a result of multimorbidity and polypharmacy as well as society and labor factors (8, 9). Unseasonal and ineffective treatments of insomnia could constitute a risk factor of physical and mental illness, substance abuse and relapse, accidents, and even mortality (10–12).

Currently, the treatments for insomnia include pharmacological and non-pharmacological measures. Although widely used, pharmacotherapy is recommended for short-term use only due to potential risks concerning hazardous side-effects, tolerance, and dependency with long-term use (13, 14). Cognitive behavioral therapy (CBT) is considered the first choice of non-prescription treatment (15), and it contains a collection of techniques (cognitive therapy, stimulus control, relaxation, sleep restriction, and hygiene) tailored to each individual, aimed at reestablishing a restful sleep (16). However, psychological therapies still remain limited and unavailable to some extent, owing to practical considerations, including financial cost and availability of resources (17). Besides this, even when patients ensure they are following CBT principles they have learned in the past, detailed questioning often reveals lapses in compliance (18).

Exercise is proposed as an alternative non-pharmacological treatment for insomnia, characterized by a safe, inexpensive, and easily accessible means of improving sleep (19). Independent of a highly specialized clinician, exercise can be completed individually or as part of a group, supervised or not, in any location, and at any time as long as it is convenient to the individual. It is reported in a randomized controlled trial (RCT) that moderate-intensity physical activity was associated with significantly reduced insomnia symptom severity and significantly elevated mood (20). Similarly, a 16-week program of moderate-intensity aerobic physical activity plus sleep hygiene education was effective in improving self-reported sleep quality, mood, and quality of life in older adults with chronic insomnia (21). Different from traditional physical exercise, mind–body exercise, characterized by gentle and slow exercise with the coordination of the body and breath (22), has received recent attention in scientific research. Varied literatures suggest that mind–body exercise (e.g., yoga, tai chi, qigong) also had beneficial effects for general health and sleep (23–25). Overall, exercise intervention shows potential superiority and promising development among non-pharmacological treatments for ameliorating insomnia and improving sleep quality, which require wider practice and application in the future.

Over the past few decades, a number of RCTs have examined the effects of exercise on sleep quality in adults (20, 23–43). However, these studies had conflicting results and no clear conclusions. We, therefore, conducted a systematic review and meta-analysis of RCTs to critically appraise the effect of exercise interventions on the sleep quality and insomnia in adults to provide a certain basis for the complementary or additional treatment of insomnia in clinical practice.

## Methods

### Data Sources and Searches

This systematic review and meta-analysis was followed by recommendations from the Preferred Reporting Items for Systematic Reviews and Meta-Analysis (PRISMA) guideline (44). A computerized literature search was carried out using PubMed, Embase, and Web of Science to identify relevant articles from inception through March 31, 2020. The search combinations were (insomnia OR sleep disorder OR sleep disturbance OR sleep insufficiency OR sleep problem OR sleep complaint) AND (exercise OR physical activity OR aerobic activity OR sport OR yoga OR tai chi OR qigong OR Pilates OR Baduanjin).

### Inclusion Criteria Based on PICOS

The PICOS framework was used to identify articles in the various databases (43, 44).

P (population) = Adult volunteers (≥18 years).
I (interventions) = Either traditional physical exercise (e.g., walking, cycling) or mind–body exercise characterized by gentle exercise with coordination of the body (e.g., yoga, tai chi), that lasted at least 2 months.
C (comparisons) = A suitable, non-physically active control group.
O (outcomes) = Validated subject or physiological measures to assess sleep quality with at least one of following measurements: PSQI, ISI, ESS rating scales and Actigraph.
S (study design) = Randomized controlled trial (RCT).

### Exclusion Criteria

(1) Investigated sleep quality in the presence of another pathological disease (e.g., cancer, Parkinson's disease, depression, obstructive sleep apnea) in participants; (2) involved atypical types of exercise (not belonging to physical or mind–body exercise), such as relaxation or music exercises; (3) examined atypical sleep regimens, such as studies with shift workers or utilizing experimentally induced insomnia; (4) involved no control group or a physically active control group only; (5) examined acute effects of exercise interventions; (6) conference abstract, observational study, dissertation, letter.

### Outcomes

We, respectively, analyzed subjective and physiological sleep outcomes. Subjective measurements contained the self-reported scoring scales of Pittsburgh Sleep Quality Index (PSQI), Insomnia Severity Index (ISI), and Epworth Sleepiness Scale (ESS). PSQI assesses different aspects of sleep quality representing the past month and is currently the most common measure of sleep quality (45, 46). ISI is a brief, reliable, and valid instrument for measuring patients' perception of insomnia in general practice and is sensitive to treatment response (47, 48). ESS is a subjective approach to evaluate daytime sleepiness and commonly used in sleep studies and clinical settings, particularly in populations with OSA (49). Physiological sleep was monitored by Actigraphy, which had four primary testing contents including sleep onset latency (SOL), total sleep time (TST), wake after sleep onset (WASO), and sleep efficiency (SE).

### Study Selection and Data Extraction

Retrieved articles were reviewed independently by three authors (YX, SL, and XJC) to choose potentially relevant articles. All disagreements on inclusion/exclusion were discussed and resolved by consensus. Two reviewers (YX and SL) independently extracted data from the included studies. The following information were extracted: the first author's name; publication year; age and gender of participants; numbers of participants in exercise and control groups, respectively; and type of exercise intervention. The information about characteristics of exercise programs included the total intervention duration (months), exercise session frequency (sessions/week), and length (minutes/session). Outcome effects contained the subjective (PSQI, ISI, ESS) and physiological (Actigraphy) measurements of sleep quality. The pre- and post-intervention means, standard deviation (SD), and number of participants in the intervention and control groups were extracted from each publication. When data was insufficient or missing, we contacted the authors for further information. The full data extraction is available in the Supplementary Material.

### Risk of Bias Assessment

The methodological quality of each trial was examined using the risk of bias criteria recommended by the Cochrane Collaboration (50). This tool evaluates the following aspects: randomness of the allocation sequence (selection bias), concealment of the allocation sequence (selection bias), blinding of participants and personnel (performance bias), blinding to outcome assessment (detection bias), incomplete outcome data (attrition bias), selective outcome reporting (reporting bias), and any other biases. Each item was described as having either a low, high, or unclear risk of bias. The scoring was carried out independently by two authors (H-HY and X-JC).

### Statistical Analysis

Effects on outcomes were expressed as the change in the measured scores from baseline to end point for exercise and control groups. If the trials did not provide changes in scores, values were calculated from scores at baseline and end point using the method recommended in the Cochrane Handbook (50). Mean differences (MDs) and 95% confidence intervals (CIs) were calculated for each study. A study including two intervention groups was split into two studies with respective exercise intervention.

We conducted subgroup meta-analyses based on the type of exercise (physical or mind–body), duration of exercise intervention (short- or long-term), gender of participants (both genders or only women), and age of participants (the elderly or the middle-aged).

Potential risk of publication bias was assessed through visual analysis of funnel plot symmetry when at least 10 trials reported the outcomes. We evaluated statistical heterogeneity using the Cochran χ^2^ test and the I^2^ statistic. For I^2^, a value of 0–25% represented insignificant heterogeneity, a value >25% but ≤50% represented low heterogeneity, a value >50% but ≤75% represented moderate heterogeneity, and a value >75% represented high heterogeneity (50). We performed fixed-effects analysis when heterogeneity was low (I^2^ ≤50%). When heterogeneity was moderate or high (I^2^>50%), we decided a priori to use random-effects analysis.

All meta-analyses were performed using Revman (version 5.3). All tests were two-tailed, and *p* < 0.05 was considered statistically significant.

## Results

### Search Results

**Figure 1:**
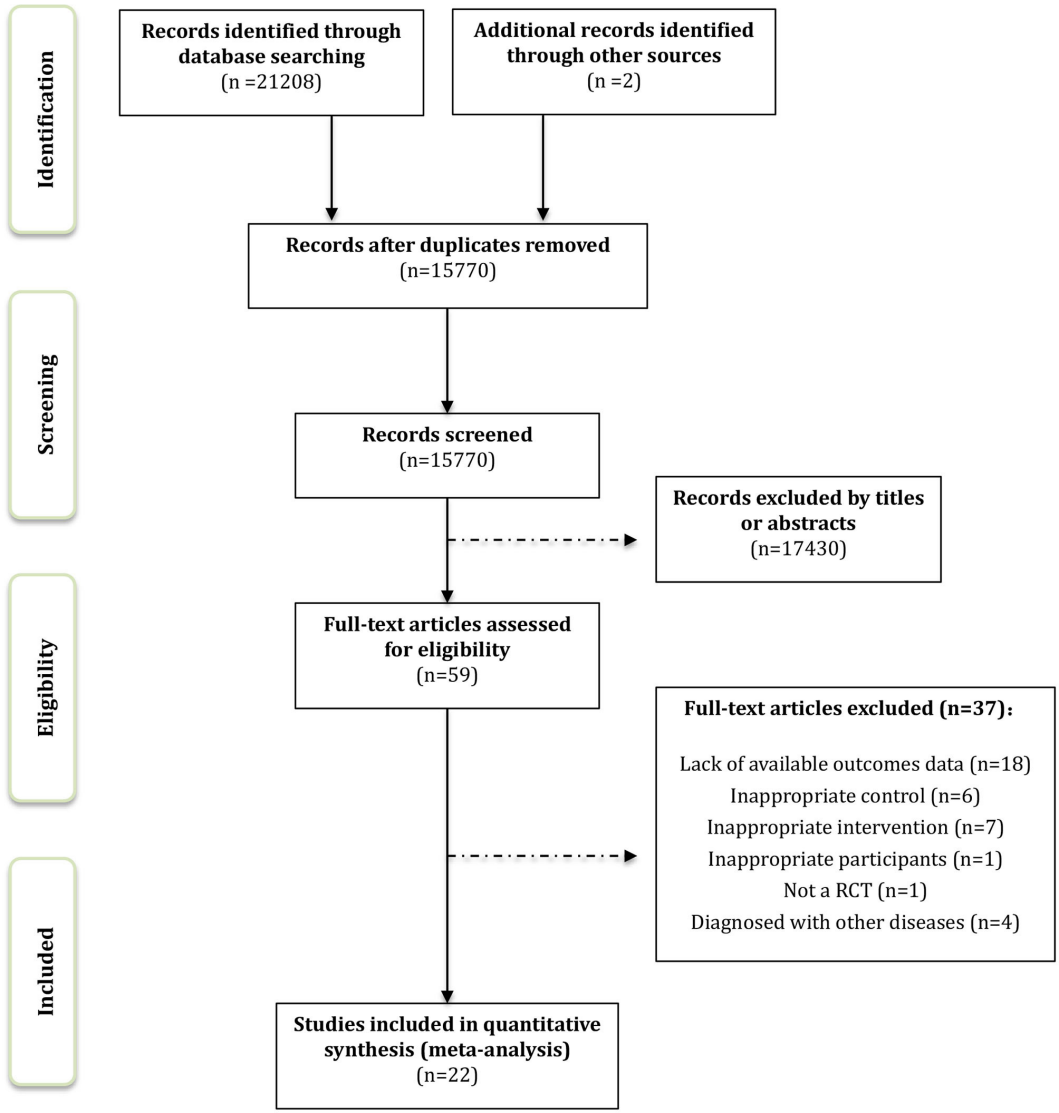
Flowchart representing the selection progress.

We identified 21,066 records through the computerized retrieval and two additional articles through the manual search. A total of 17,489 articles were retained by removing duplicates. After screening titles and abstracts, 17,430 articles were excluded. The full texts of 59 remaining articles were reviewed for inclusion, and finally, 22 RCTs were considered eligible for systematic review and meta-analysis. Details of the study selection process are shown in Figure 1.

### Characteristics of the Studies

We included 22 randomized control trials in the mate-analysis (20, 23–43). All studies were published between 1995 and 2019, involving 1,806 participants. The majority of studies included mixed-gender groups, seven studies focused on women only and, of these, one involved post-partum women (34), and five involved peri- or post-menopausal women (24, 25, 38, 39, 43). For exercise types, 13 trials performed physical exercise, and 11 trials performed mind–body exercise. Among these, two trials included both physical and mind–body exercise groups (39, 40). Total duration of exercise programs ranged from 2 to 12 months with respective frequency and length of sessions. Of these 22 articles, subjective sleep quality was assessed in 14 studies with the PSQI scale, in four studied with the ISI scale (20, 25, 27, 43), and in two studies with the ESS scale (28, 33). Six studies evaluated physiological sleep conditions by the use of Actigraphy (27–29, 35, 39, 41). Details of study characteristics are presented in Table 1.

**Table 1:** Characteristics of the included trials and participants.

**Included studies**	**Participants gender**	**Participants' age (years)**	**Sample size (N)**	**Intervention**	**Intervention duration (months)**	**Session duration/frequency**	**Subjective outcomes**	**Physiological outcomes**
Karimi et al. (26)	Men	≥60	IG = 23; CG = 23	Walking	2	30 min, 3 times/week	PSQI	–
Yeung et al. (27)	Both	18–65	IG = 18; CG = 19	Physical exercise training (ZTEx)	2	2 h, twice/week	ISI	Actigraph
Tan et al. (28)	Men	30–65	IG = 24; CG = 21	Aerobic exercise	6	30–60 min, 1–5 times/week	ESS	Actigraph
Chen et al. (29)	Both	55–70	IG = 33; CG = 34	Aquatic exercise	2	60 min, twice/week	–	Actigraph
Irwin et al. (30)	Both	59–86	IG = 59; CG = 53	Tai Chi	4	40 min, 3 times/week	PSQI	–
Irwin et al. (23)	Both	≥55	IG = 48; CG = 25	Tai Chi	4	2 h, once/week	PSQI	–
Afonso et al. (25)	Women	50–65	IG = 15; CG = 15	Yoga	4	1 h, twice/week	ISI	–
Hartescu et al. (20)	Both	≥40	IG = 17; CG = 19	Walking	6	≥ 30 min, ≥ 5 times/week	ISI	–
King et al. (31)	Both	≥55	IG = 36; CG = 30	Aerobic exercise	12	60 min, 5 times/week	PSQI	–
King et al. (32)	Both	50–76	IG = 20; CG = 23	Aerobic exercise	4	60 min, 4 times/week	PSQI	–
Brandão et al. (33)	Both	≥60	IG = 64; CG = 61	Physical exercise	3	40 min, ≥3 times/week	PSQI ESS	–
Ashrafinia et al. (34)	Women	18–35	IG = 40; CG = 40	Pilates	2	30 min, 5 times/week	PSQI	–
Alessi et al. (35)	Both	≥65	IG = 33; CG = 32	Physical exercise	2.25	2 h, 5 times/week	–	Actigraph
Chen et al. (36)	Both	≥60	IG = 27; CG = 28	Baduanjin	3	30 min, 3 times/week	PSQI	–
Chen et al. (37)	Both	≥65	IG = 59; CG = 55	Wheelchair-bound senior elastic band exercise	6	40 min, 3 times/week	PSQI	–
Aibar-Almazan et al. (38)	Women	≥60	IG = 55; CG = 52	Pilates	3	60 min, twice/week	PSQI	–
Yeh and Chang (24)	Women	≥45	IG = 35; CG = 35	Qigong	3	30 min, once/week	PSQI	–
Buchanan et al. (39)	Women	40–62	IG1 = 52; IG2 = 54; CG = 80	IG1:Yoga; IG2:Aerobic exercise	3	IG1:90 min, once/week; IG2:40–60 min, 3 times/week	–	Actigraph
Elavsky and McAuley (40)	Women	42–58	IG1 = 63; IG2 = 61; CG = 39	IG1:Walking; IG2:Yoga	4	IG1:60 min, 3 times/week; IG2:90 min, twice/week	PSQI	–
Oudegeest-Sander et al. (41)	Both	≥65	IG = 11; CG = 10	Cycling	12	35 min, 3 times/week	–	Actigraph
Chen et al. (42)	Both	≥65	IG = 31; CG = 24	Yoga	6	70 min, 3 times/week	PSQI	–
Sternfeld et al. (43)	Women	40–62	IG = 79; CG = 131	Aerobic exercise	3	40–60 min, 3 times/week	PSQI ISI	–

*IG, intervention group; CG, control group*.

### Quality Evaluation

The risk of bias summary for all studies included in the meta-analysis can be found in Supplementary Figure 1. Supplementary Figure 2 shows the risk of bias for self-reported and physiological measurement of each RCT according to the Cochrane risk of bias tool (50). Of these 22 trials, 12 trials described an adequate random sequence generation process, and five trials described the methods used for allocation concealment. As blinding of participants was not possible for exercise intervention (exercise vs. no exercise), the assessments for performance bias in all studies were high risks. Regarding blinding of outcome assessment, we judged studies separately for physiological and self-reported outcomes; thus, not 100% of studies had both these assessments. Blinding of self-reported outcomes assessments is not achievable in practice, and only studies with a physiological measurement (Actigraph) could be rated at low risk.

**Figure 2:**
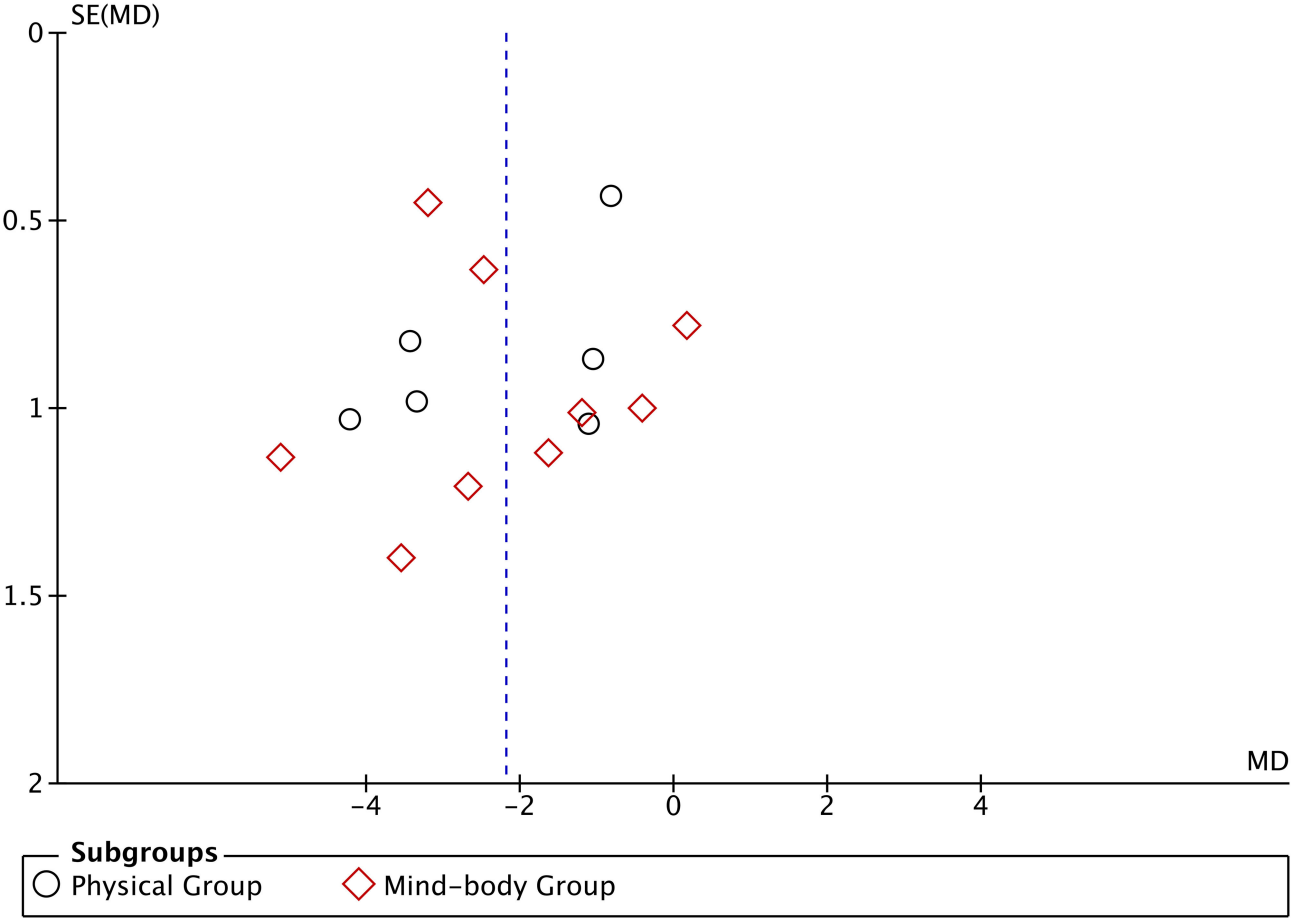
Funnel plot assessing the publication bias.

We used a funnel plot to check for potential publication bias (Figure 2). No obvious asymmetry was identified in the funnel plot, indicating that publication bias was unlikely.

### Effects of Exercise on Sleep Quality in Subjective Outcomes

**Figure 3:**
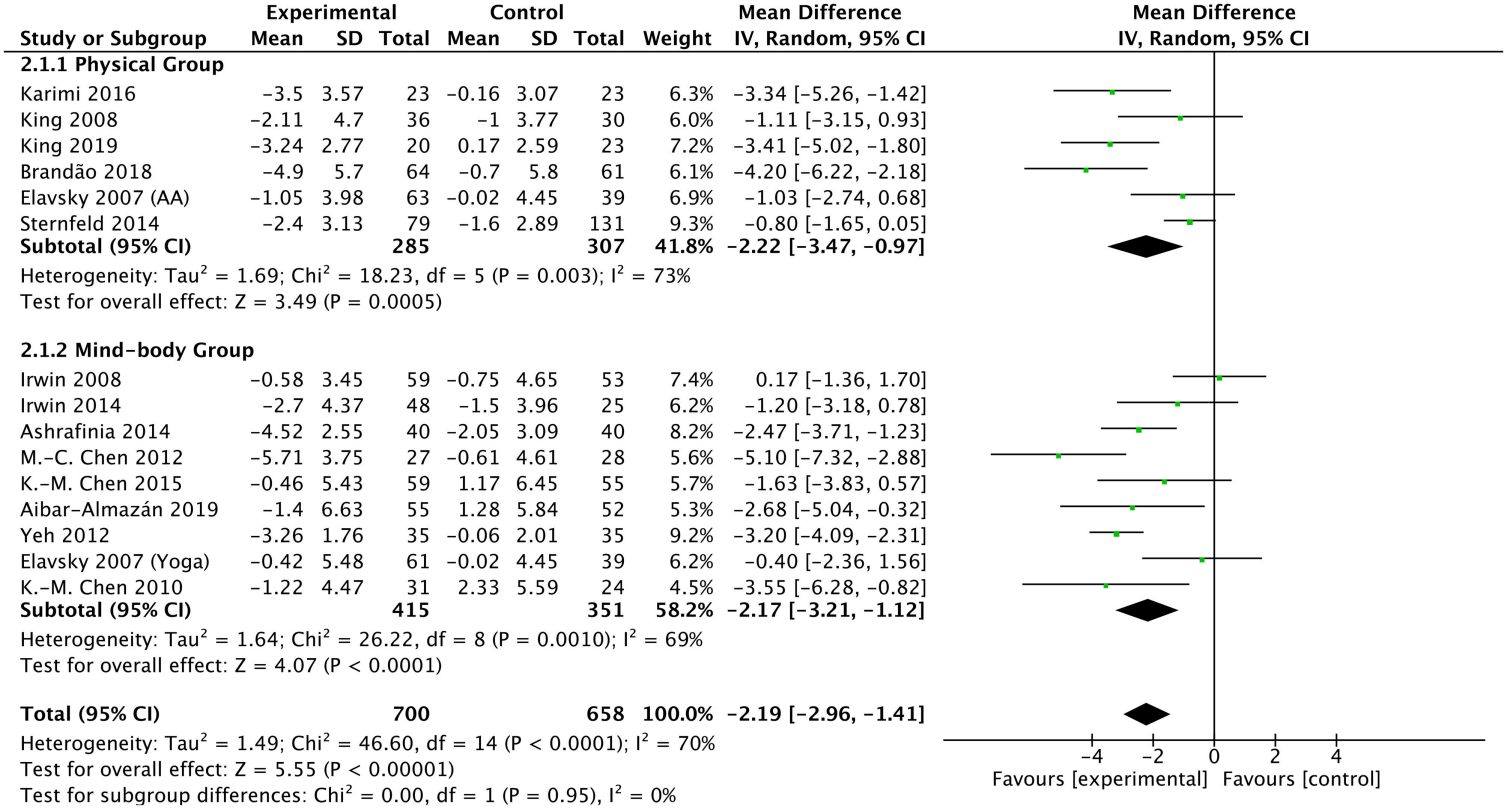
Forest plot of PSQI scale outcomes in overall analysis and subgroup analysis stratified by the type of exercise intervention.

A total of 14 studies were included in the meta-analysis for the PSQI measurements. The pooling results of these studies suggest that exercise had a statistically significant effect on sleep quality vs. control (a larger reduction in PSQI score) with a pooled MD of −2.19 (95% CI −2.96 to −1.41, *p* < 0.00001) (Figure 3). Heterogeneity among studies was found to be moderate (X^2^ = 46.6, df = 14, *p* < 0.00001, I^2^ = 70%).

**Figure 4:**
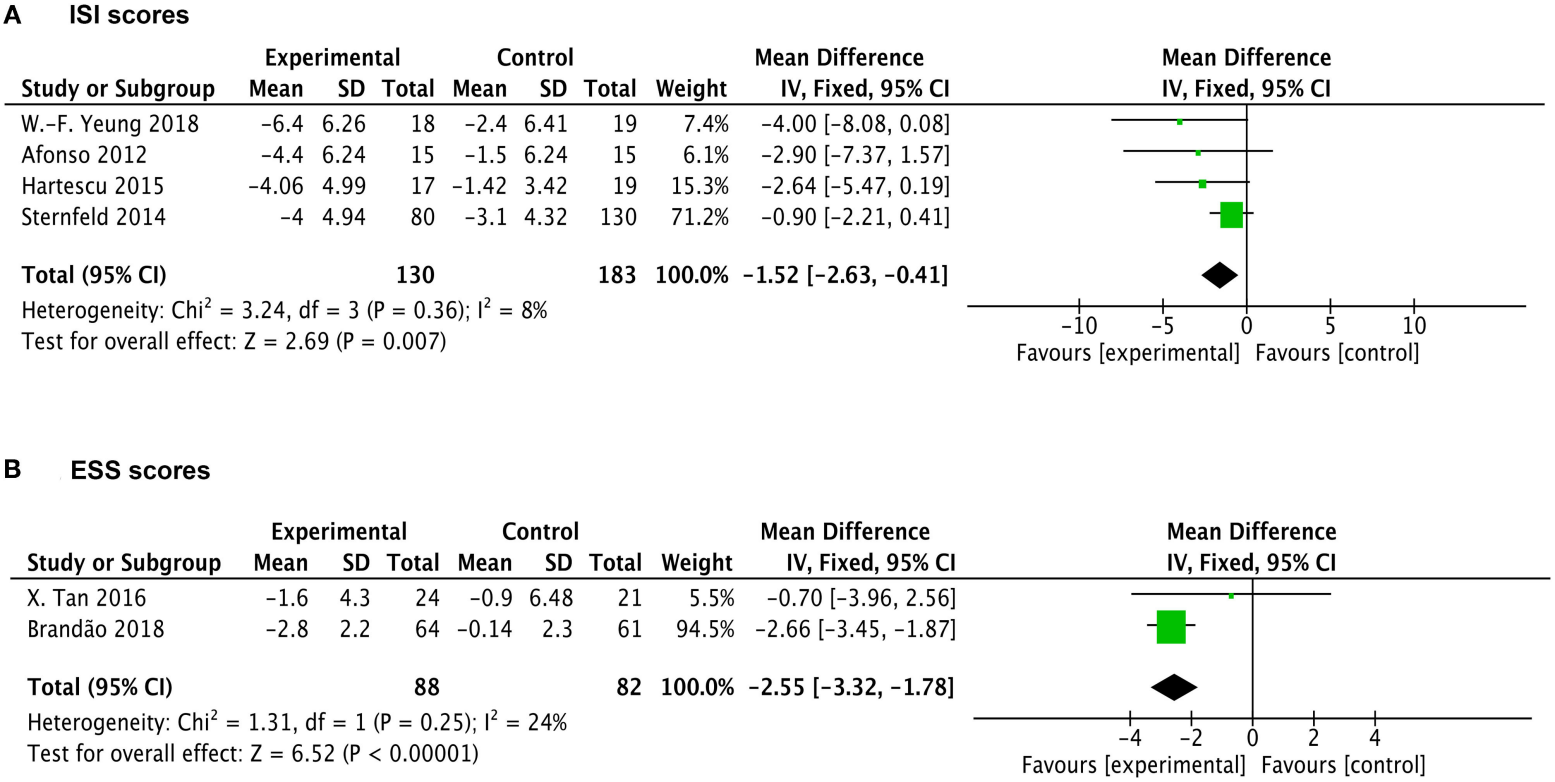
Forest plots of ISI and ESS scale outcomes in overall analysis. **(A)** Forest plot of ISI scale outcomes. **(B)** Forest plot of ESS scale outcomes.

Four studies reported the outcomes of ISI measurements. As shown in Figure 4A, analysis indicates significant reduction of ISI score in the exercise group compared with the control group with a pooling MD of −1.52 (95% CI −2.63 to −0.41, *p* = 0.007). The heterogeneity among studies was found to be low (X^2^ = 3.24, df = 3, *p* = 0.36, I^2^ = 8%). Two articles provided the evaluating outcomes of the ESS scale. The exercise group demonstrated evident decreased ESS scores vs. the control group with a pooling MD of −2.55 (95% CI −3.32 to −1.78, *p* < 0.00001) (Figure 4B). Heterogeneity among studies was found to be low (X^2^ = 1.31, df = 1, *p* = 0.25, I^2^ = 24%). Of these, one study showed high weight in the analysis due to a relatively large sample size and narrow confidence interval. Taking it into account, the exercise intervention still resulted in improvements of sleep quality in ESS scoring.

### Subgroup Analysis

We further performed subgroup analyses of PSQI measurements in 14 studies based on the following variables: the type of exercise (physical or mind–body), duration of exercise intervention (short- or long-term), gender of participants (both genders or only women), and age of participants (the elderly or the middle-aged). Regarding the exercise type, physical exercise interventions (MD −2.22; 95% CI −3.47 to −0.97; *p* = 0.0005) and mind–body exercise interventions (MD −2.17; 95% CI −3.21 to −1.12; *p* < 0.0001) both had favorable effects on sleep disturbance (Figure 3, Table 2). There was no significant difference of enhancing effects between physical exercise and mind–body exercise (*p* = 0.95).

**Table 2:** Subgroup analysis bases on exercise type, exercise duration, participants' sex, participants' age.

**Variable**	**No. of trials**	**No. of participants**	**Pooling estimates**		**I^**2**^**	**P_**differences**_**
			**MD (95%CI)**	***P-*value**		
**Type of exercise**						
Physical exercise	6	592	−2.22 [−3.47, −0.97]	0.0005	73%	0.95
Mind-body exercise	9	766	−2.17 [−3.21, −1.12]	0.001	69%	
**Duration of exercise**						
Short-term (≤3 months)	7	693	−2.95 [−4.08, −1.81]	<0.00001	77%	0.04
Long-term (>3 months)	8	665	−1.42 [−2.37, −0.47]	0.003	49%	
**Gender of participants**						
Women and men	8	643	−2.43 [−3.76, −1.10]	0.0003	72%	0.47
Women only	6	669	−1.80 [−2.86, −0.73]	0.0009	74%	
**Age of participants**						
The elderly	10	796	−2.54 [−3.62, −1.45]	<0.00001	66%	0.30
The middle-aged	5	562	−1.68 [−2.86, −0.50]	0.0009	69%	

*MD, mean difference; CI, confidence interval*.

Considering subgroups by duration of exercise intervention, short-term (≤3 months) interventions (MD −2.95; 95% CI −4.08 to −1.81; *p* < 0.00001) had somewhat stronger benefits on sleep than longer term (>3 months) interventions (MD −1.42; 95% CI −2.37 to −0.47; *p* = 0.003) (Table 2). The difference between these two subgroups was statistically significant (*p* = 0.04).

Regarding gender of participants, the results showed beneficial improvements of sleep quality in trials involving both men and women (MD −2.43; 95% CI −3.76 to −1.1; *p* = 0.0003) and trials involving women only (MD −1.8; 95% CI −2.86 to −0.73; *p* = 0.0009) (Table 2). Subgroup analysis of male participants only was excluded owing to just one study being eligible. Additionally, we performed subanalysis based on age of participants. As shown in Table 2, the exercise intervention seemed to have potentially larger benefit on the sleep quality in the elderly (MD −2.54; 95%CI −3.62 to −1.45; *p* < 0.00001) compared with the middle-aged (MD −1.68; 95% CI −2.86 to −0.50; *p* = 0.005) although this observed tendency was not statistically significant (*p* = 0.3).

### Effects of Exercise on Sleep Quality in Physiological Outcomes

**Figure 5:**
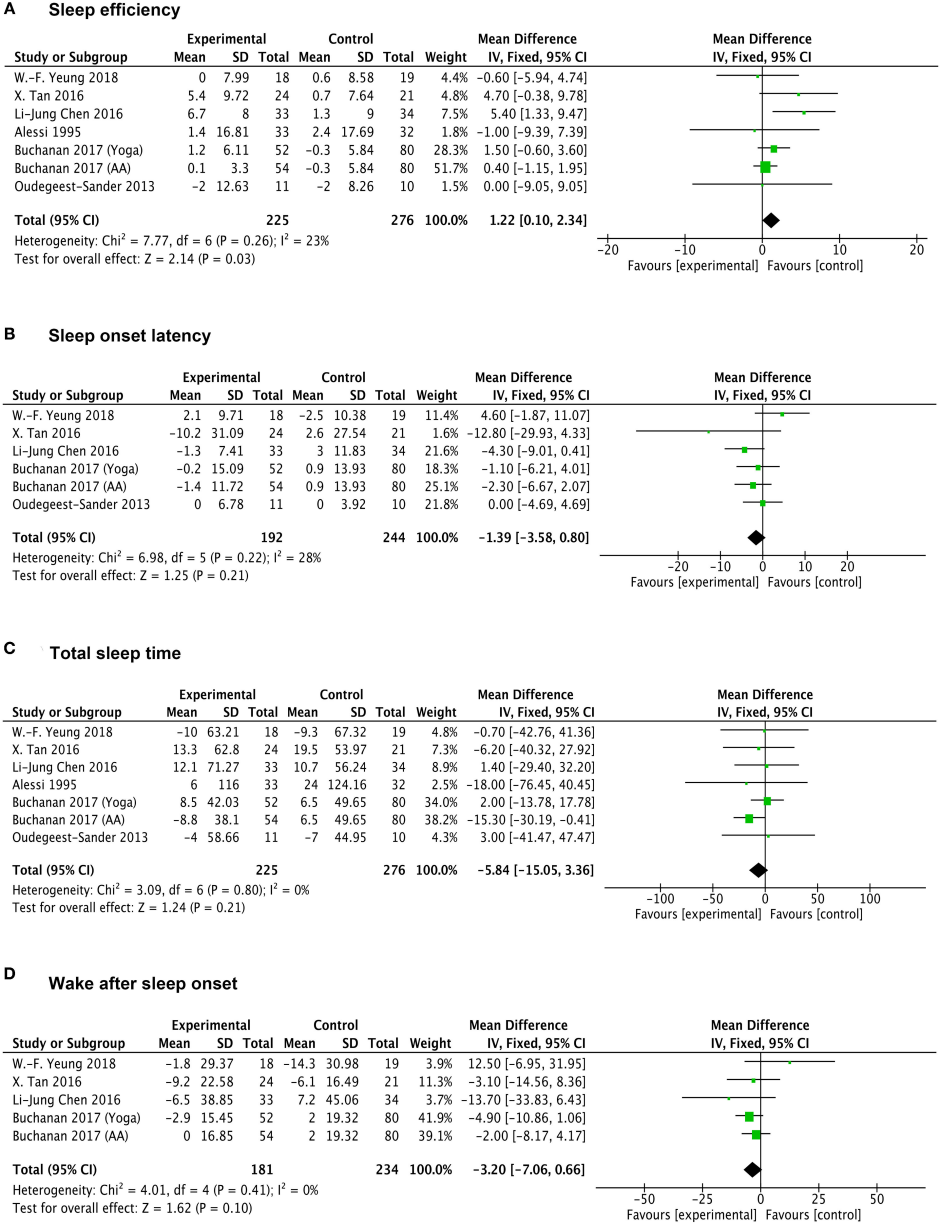
Forests plot of Actigraph monitoring outcomes in overall analysis. **(A)** Forest plot of sleep efficiency outcomes. **(B–D)** Forest plot of sleep onset latency, total sleep time, wake after sleep onset, respectively.

A total of six studies reported the data measured by Actigraph to evaluate the physiological sleep in participants. Of these, one trial contained two different exercise interventions (aerobic activity and yoga), one trial lacked the data of WASO, two trials lacked the data of SOL and WASO. Interestingly, we found a significantly favorable effect of exercise on physiological SE (MD 1.22; 95% CI 0.1 to 2.34; *p* = 0.03; I^2^ = 23%) in participants vs. control intervention (Figure 5A). Other results show that, the participants under exercise intervention tended to have mild decreased SOL (MD −1.39; 95% CI −3.58 to 0.8; *p* = 0.21; I^2^ = 28%) and total sleep time (MD −5.84; 95%CI −15.05 to 3.36; *p* = 0.21; I^2^ = 0%) as well as less time of WASO (MD −3.2; 95%CI −7.06 to 0.66; *p* = 0.1; I^2^ = 0%) compared with the control intervention (Figures 5B–D). However, these differences between two groups were all not statistically significant.

## Discussion

This systematic review and meta-analysis focused on RCTs to examine the effects of physical and mind–body exercises on sleep quality and insomnia in adults. Inclusion criteria were strictly set and applied to ensure that only adults without other clinical diseases were included. According to 22 eligible trials, we extracted and analyzed subjective sleep (PQSI, ISI, ESS scales) and physiological sleep (Actigraphy) outcomes to evaluate the sleep quality change after exercise interventions.

The overall analyses suggest evident improvements of exercise on self-reported sleep quality in adults. Significantly larger reductions in sleep rating scales (PSQI, ISI, and ESS) were found in the exercise groups with low-to-moderate heterogeneity among studies. Specifically, exercise had positive effects on sleep quality and poor sleep indicated by reduced PSQI scores. As well, insomnia severity in participants was partially relieved after intervention of exercise, presented by lower ISI scores. Compensatory daytime sleepiness, often resulting from poor sleep in the night, could also be improved by regular exercise with decreased ESS rating.

In the subgroup analyses of PSQI scores, our quantitative evaluation indicates that both physical and mind–body exercise could improve sleep in participants. However, results did not reveal an obvious superiority of specific exercise type (physical or mind–body) on sleep problems. We did not perform more detailed classification of exercise types (e.g., walking, swimming, cycling, yoga, tai chi) to carry out quantitative analysis due to a small number of studies for each item. It was reported that mind–body exercise might have a different or additional mechanistic route to sleep improvement beyond that of traditional physical exercise (13, 51).

Besides this, we examined the effects of subgroups sorted by duration of exercise intervention, gender, and age of participants in PSQI scores. Short-term (≤3 months) exercise seemed more effective for betterment of sleep disturbance compared with long-term (>3 months) exercise in the subgroup analysis. Given the variables of exercise types, it could not be judged that short-term exercise was more beneficial to sleep than long-term exercise. In one study with an intervention of cycling exercise, the exercise group demonstrated less SOL and increasing SE in the 6 month compared to the 12th month monitored by Actigraphy (41). The results of another trial involving yoga intervention indicated that participants had better self-reported sleep in the 6 month than the third month in PSQI scores (42). It was supposed that mind–body exercise might need a relatively longer intervention time to promote sleep quality compared with physical exercise, which provides references for us to choose adequate exercise type and duration. For gender of participants, there was no difference of sleep-improving function by exercise among trials containing women only and trials containing two genders. For subanalysis stratified by age of participants, exercise intervention showed significant effects on sleep disturbance in the middle aged. Although, in the elderly group, a tendency of larger relief was found although it was not statistically significant. In practice, sleep becomes more fragmented with age, characterized by the increased number of awakenings as well as the time spent awake at night (52, 53). Strong predictors of poor sleep in the elderly included mood disorders and physical illness (54, 55). It was postulated that the elderly were more susceptible to exercise intervention due to its mood-relief affects.

Physiological sleep quality was evaluated by use of Actigraphy, which is applied as the best choice for physiological measurement because polysomnography (PSG) is very expensive and difficult to implement (56). Quantitative analysis illustrates significantly increased SE in exercise groups vs. control groups. No significant differences were found in the items of SOL, TST, and WASO between groups from included studies although there existed mild tendencies of decreased SOL, TST, and WASO after exercise intervention. Despite evident effects being observed for subjective sleep outcomes, exercise was not shown to effectively improve physiological parameters of sleep in our meta-analysis. It was reported that, as validated questionnaires measured additional constructs that contributed to overall sleep quality (e.g., daytime impairment), exercise could potentially exert its effects through these constructs rather than impact sleep directly (13). This might partially explain the conflicting results between the physiological and subjective measures of sleep in individuals. Further mechanical explorations of subjective and physiological sleep could help to explain this conflict on a theoretical basis.

CBT has been commonly considered as the preferred treatment of insomnia (57). Two published studies compared the effects of exercise intervention and CBT on sleep quality in cancer patients. The results illustrated that both interventions produced clinically meaningful improvements of sleep; however, exercise was not significantly superior to CBT (58, 59). It was proposed that exercise could function as a complementary or additional method to ameliorate the sleep condition among people, playing a joint role with CBT (58, 59).

In clinical practice, exercise intervention could be achieved in many ways: at home or outside, spontaneous or supervised, at any time convenient to the individuals. Remarkably, exercise intervention should be implemented chronically on a regular basis, such as three to five times a week with a certain duration of each time. A published trial reported that just 1 day of moderate-to-vigorous exercise compared with no-exercise did not facilitate sleep need or alter other sleep measures other than the amount of light sleep (60). Nowadays, increasing number os exercise apps emerge, which could generally record and monitor our exercise-related information, such as exercise duration and frequency, as well as our real-time heart rate and breath frequency, etc. (61, 62). These records serve as encouragement to patients and a reflection of patients' compliance with the clinician.

We acknowledge several limitations on this meta-analysis involving various factors. (1) Risk of bias was high in all included studies, related to lack of blinding to participants or personnel. (2) Heterogeneity of effects was moderate across studies in analysis of PSQI scales, possibly associated with insomnia prevalence also varying with ethnicity and obesity status (63). (3) Additionally, it is of certain significance to analyze the follow-up data after the finish of exercise intervention, which could reveal the longer-term effects of regular exercise. Due to a lack of complete and effective records about follow-up information in existing articles, we did not perform this part of the analysis in the study, which could be improved afterward. (4) The content of the control group appeared to influence outcomes with larger group differences observed in studies utilizing a wait-list control group, receiving no form of intervention or contact. It could be hypothesized that for those studies whereby the control group did receive some form of intervention (e.g., sleep seminar education, sleep hygiene, group recreational activities), participants gained some benefit through non-specific means. Alternatively, it might be that wait-list controls operated similar to nocebo conditions (13, 64).

To the author's knowledge, this is the first meta-analysis research to investigate the sleep-enhancing effects of regular exercise in adults without other specific diseases (e.g., cancer, depression, etc.), involving both subjective and physiological sleep outcomes. Two published meta-analysis articles, respectively, explored the effects of exercise only on middle-aged women (65) and pregnant women (66). Some other meta-analysis studies assessed whether exercise improves sleep in adults who already have a diagnosis of insomnia (67, 68). One of them appraised several alternative interventions for participants with insomnia, including exercise, melatonin, and others. They found exercise was efficacious in ameliorating self-reported SOL with small-to-medium effects. However, the number of eligible studies for exercise intervention is still small to confirm the above conclusion (68). Herein, our study focused on the sleep-enhancing effects of exercise on the whole adult population, not limiting the initial sleep conditions of participants as the participants in most RCTs were not necessarily all diagnosed with insomnia but they were still experiencing or susceptible to poor sleep conditions. We further performed subgroup analyses based on the variables of exercise types and duration, participants' age and gender to explore more detailed links between exercise and sleep quality.

In conclusion, our systematic review and meta-analysis suggest that regular exercise exerts beneficial effects on sleep quality and insomnia in adults, primarily in subjective sleep measurements. Exercise could potentially serve as a complementary and additional therapy of pharmacological measures and CBT therapy for treatments of chronic insomnia. Varied physical and mind–body exercise should be encouraged to perform in all age groups to relieve sleep problems.

## Data Availability Statement

The original contributions presented in the study are included in the article/Supplementary Materials, further inquiries can be directed to the corresponding author/s.

## Author Contributions

YX, SL, YY, and WW designed the study and wrote the protocol. Independent screening and data extraction of eligible studies was conducted by YX, SL, and X-JC. The studies' quality scoring was carried out independently by H-HY and X-JC. YX and SL conducted the statistical analysis and wrote the first draft of the manuscript. WW and YY critically revised the manuscript for important intellectual content. All authors contributed to and have approved the final manuscript.

## Conflict of Interest

The authors declare that the research was conducted in the absence of any commercial or financial relationships that could be construed as a potential conflict of interest.

## Supplementary Material

Supplementary Figure 1 Risk of bias summary.

Supplementary Figure 2 Risk of bias assessments: –, low risk of bias; +, high risk of bias; ?, unclear risk of bias.
