# Supplementary figures and images for: Effects of Exercise on Sleep Quality and Insomnia in Adults: A Systematic Review and Meta-Analysis of Randomized Controlled Trials
Source: Front Psychiatry. 2021 Jun 7;12:664499. doi: 10.3389/fpsyt.2021.664499 (PMC8215288; doi:10.3389/fpsyt.2021.664499)

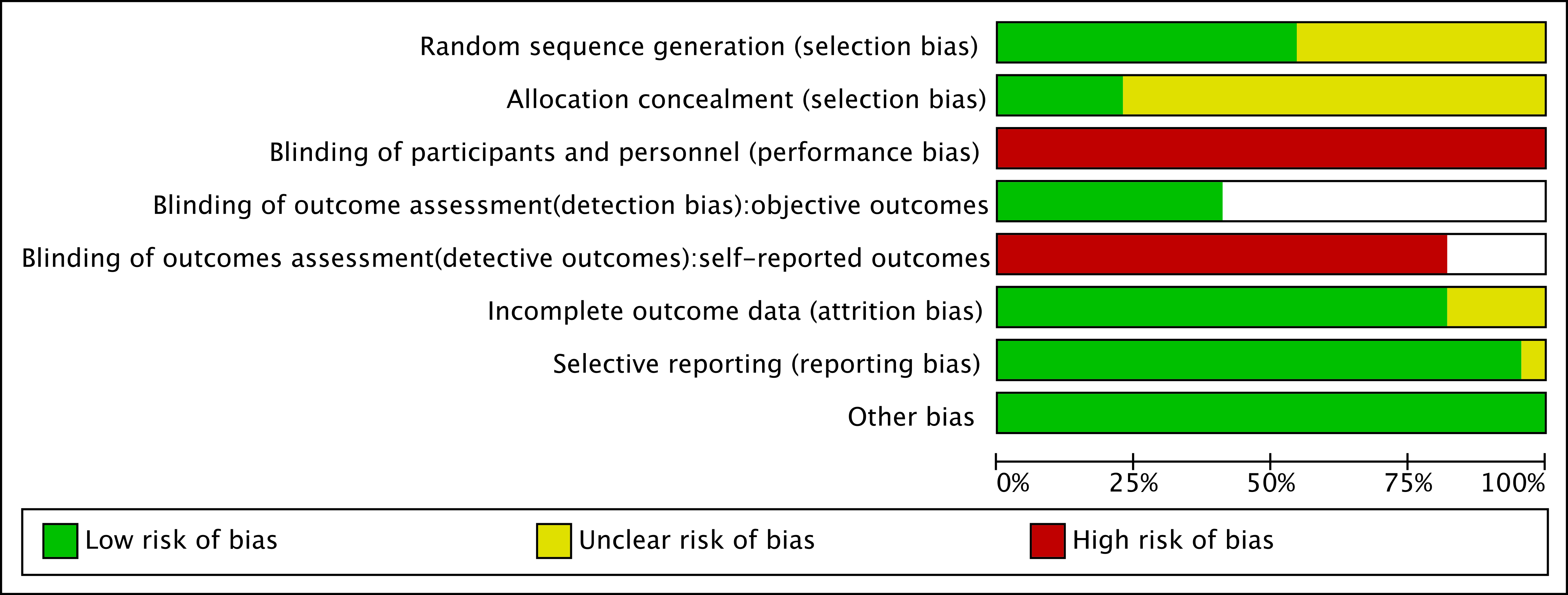

Supplement: Supplementary Figure 1 — Risk of bias summary. [file Image_1.TIF]

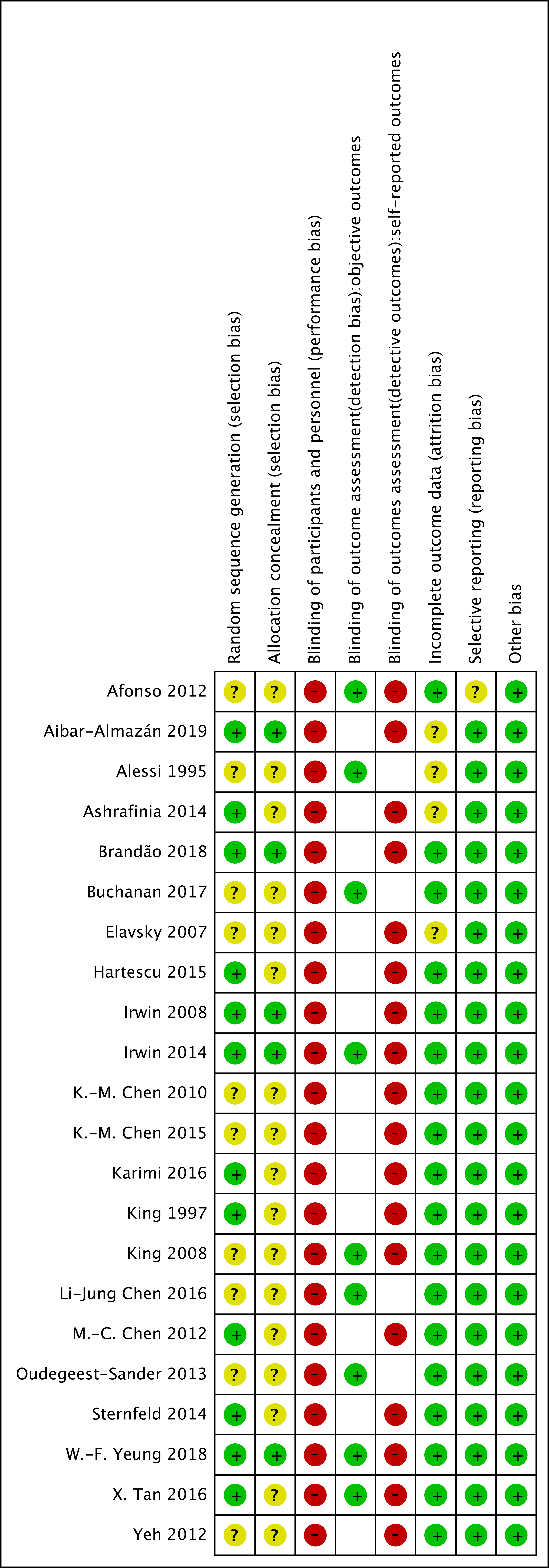

Supplement: Supplementary Figure 2 — Risk of bias assessments: –, low risk of bias; +, high risk of bias; ?, unclear risk of bias. [file Image_2.TIF]
